# Promoting equity, diversity, and inclusion in regional anesthesia academic publishing: a call to action

**DOI:** 10.1186/s44158-024-00145-7

**Published:** 2024-02-12

**Authors:** Francesca Rubulotta, Thomas M. Hemmerling, Sahar Bahrami

**Affiliations:** 1 Department of Critical Care Medicine, McGill University, Montreal, Canada; 2 IWIN Foundation, Vittorio Emanuele 329 Agira, Sicily, Italy; 3 International, Society of Technology in Anesthesia, Research, Society of Technology in Anesthesia, Department of Anesthesia, Fleury Hospital — CSSS Ahuntsic and Montreal-Nord, McGill University & Biomedical Engineering, UdM, Montreal, Canada

The world is not on track to achieve gender equality by 2030. The recently published manuscript, “Examining Gender Bias in Regional Anesthesia Academic Publishing: A 50-year Bibliometric Analysis,” sheds light on a pressing issue within academic publishing [1]. The study’s findings highlight the need for increased representation and opportunities for women in the field of regional anesthesia, raising important questions about gender bias [2]. Therefore, academic publishing plays a pivotal role in disseminating knowledge; shaping scientific discourse, education, and training; and informing medical practice. This editorial aims to comment on the significance of these findings and emphasize the urgent need to address globally gender bias in academic publishing.

The study’s bibliometric analysis reveals a detectable underrepresentation of women in regional anesthesia research (women first authors 19.3%, last authors 15.5%, and single author 10.6%) [1], which suggests that gender impartiality may hinder their chances of academic recognition and advancement. The journal called “Regional Anesthesia and Pain Medicine” (RAPM) is a respected journal in this field. However, looking at their publication record since 1976 is not an exhaustive gender examination in “regional anesthesia academic publishing [1].” Moreover, most anesthesia journals have a section on regional anesthesia, so complete examination of gender bias in “regional anesthesia academic publishing” should expand to investigate all publications in this “academic area” which is a challenge!

Women do not publish as much as their male counterparts; regardless, there are more women in medicine nowadays than men [2]. The literature has largely described a persistent gender publication gap among academic physicians [3]. The positive trend in closing this gap is noticeable in the last decade in all medical journals. All specialties rate of female first authors among six prominent medical journals in the USA raised from 5.9% in 1970 to 29.3% in 2004; the rate of female last authors in the same journals raised from 3.7 to 19.3%, respectively [3]. Women anesthesiologists in general have a lower h-index compared to men (4.72 vs 9.49 among professors), even after adjustment for faculty rank [4]. This associates frequently to the fact that women are also underrepresented in meetings (as speakers and chairs), editorial boards of journals, and scholarly societies [2, 5]. In general, women are more likely to publish in a lower-impact factor journal than men [6]. The gender gap in Hirsch index (h-index) is considered for career advancement or hiring [7, 8]. Journals are trying to ensure diverse representation in their editorial boards [9, 10]. Currently, women represent 9% of editorial boards in the orthopedic journals with the highest impact factors, 21% of general surgery journals (*p* < 0.001), and 35% of internal medicine journals (*p* < 0.001) [9]. Women constitute 6% of editorial board members in the *Canadian Journal of Anesthesia*: from 1954 to 2018 [11]. It is reasonable to believe that the basis of gender disparities leading to a significant gap is complex and multifactorial. Among leading causes are limited networking abilities at early stages of career [5] and the lack of sponsorship and mentorship [12]. Networking is crucial to career growth as it involves meeting people at conferences, courses, and educational events. Good networking can mean the difference between a “mediocre and a great career [5].” 

Recently, all junior doctors (female and male) wish to focus on other objectives in life, mainly on having children and starting a family. Therefore, these reflections apply for men at early career stages, who have done a bit of research before and loose opportunities at the time they start a family or have children.

To tackle gender bias in academic publishing, society must first acknowledge its existence and the impact it has on the careers of women and junior doctors. The academic community, research institutions, publishing houses, and funding agencies all have a responsibility to actively strive for equity and inclusion. Diverse perspectives foster creativity, critical thinking, and the generation of transformative ideas. By ensuring this diversity of representation, journals demonstrate a conscious commitment to work against unintentional promotion of one view or perspective at the exclusion of others, which can result in disengaging individuals and reducing participation by diverse key players [2, 9].

This is the reason many journals are engaging and working hard to achieve and maintain equity by proactively increasing representation and providing a range of opportunities for others to lead and participate in key decision-making [9]. There are many barriers to diversity: language, lack of skills and knowledge, lack of information about the selection process, and the mechanism. One way this could be solved is by creating a database of researchers from different countries and allowing researchers to publish in dual/several languages. Increasing representation and opportunities for women and minorities in regional anesthesia academia requires a multifaceted approach. Mentorship programs, scholarships, and dedicated funding for women in research can help remove barriers and provide valuable support for their careers. Moreover, sponsorship conferences and research organizations should strive to achieve gender balance in speaker lineups and leadership positions.

Publishers must adopt policies that promote transparency and fairness, ensuring that the quality of research is the primary basis for publication, rather than the gender or affiliation of the authors. Peer review processes should be designed to minimize bias, considering anonymity and a diverse pool of reviewers.

## Conclusion

The underrepresentation of women indicates disparities in regional anesthesia academic publishing, which is not just a matter of equality; it is an obstacle to progress and to innovation within regional anesthesia and other medical specialties [1, 2]. The manuscript on gender bias in regional anesthesia academic publishing illuminates a critical issue that demands immediate attention. Achieving inclusion, equity, and diversity in academic publishing is not only a matter of fairness and justice but a means to foster a diverse and vibrant research community. By addressing gender bias and providing greater representation and opportunities for women, we can unlock new perspectives, enhance innovation, and ultimately advance the field of regional anesthesia. It is time for all stakeholders to come together and act in dismantling gender barriers, creating a more inclusive future in academic publishing.

## Data Availability

Not applicable.
